# Metabolic intermediate acetyl phosphate modulates bacterial virulence *via* acetylation

**DOI:** 10.1080/22221751.2018.1558963

**Published:** 2019-01-16

**Authors:** Jie Ren, Yu Sang, Ran Qin, Yang Su, Zhongli Cui, Zhiguo Mang, Hao Li, Shaoyong Lu, Jian Zhang, Sen Cheng, Xiaoyun Liu, Jixi Li, Jie Lu, Wenjuan Wu, Guo-Ping Zhao, Feng Shao, Yu-Feng Yao

**Affiliations:** a Laboratory of Bacterial Pathogenesis, Department of Microbiology and Immunology, Institutes of Medical Sciences, Shanghai Jiao Tong University School of Medicine, Shanghai, People’s Republic of China; b Key Laboratory of Agricultural Environmental Microbiology, Ministry of Agriculture, College of Life Sciences, Nanjing Agricultural University, Nanjing, People’s Republic of China; c Department of Pharmaceutical Science, School of Pharmacy, East China University of Science & Technology, Shanghai, People’s Republic of China; d Department of Pathophysiology, Key Laboratory of Cell Differentiation and Apoptosis of Chinese Ministry of Education, Shanghai Jiao Tong University School of Medicine, Shanghai, People’s Republic of China; e Institute of Analytical Chemistry and Synthetic and Functional Biomolecules Center, College of Chemistry and Molecular Engineering, Peking University, Beijing, People’s Republic of China; f State Key Laboratory of Genetic Engineering, Collaborative Innovation Center of Genetics and Development, Shanghai Engineering Research Center of Industrial Microorganisms, School of Life Sciences, Fudan University, Shanghai, People’s Republic of China; g Department of Infectious Diseases, Shanghai Ruijin Hospital, Shanghai, People’s Republic of China; h Department of Laboratory Medicine, Shanghai East Hospital, Tongji University School of Medicine, Shanghai, People’s Republic of China; i Key Laboratory of Synthetic Biology, Institute of Plant Physiology and Ecology, Shanghai Institutes for Biological Sciences, Chinese Academy of Sciences, Shanghai, People’s Republic of China; j National Institute of Biological Sciences, Beijing, People’s Republic of China

**Keywords:** Acetyl phosphate, acetylation, phosphorylation, PhoP, virulence, metabolism

## Abstract

Accumulating evidence indicates that bacterial metabolism plays an important role in virulence. Acetyl phosphate (AcP), the high-energy intermediate of the phosphotransacetylase-acetate kinase pathway, is the major acetyl donor in *E. coli*. PhoP is an essential transcription factor for bacterial virulence. Here, we show in *Salmonella typhimurium* that PhoP is non-enzymatically acetylated by AcP, which modifies its transcriptional activity, demonstrating that the acetylation of Lysine 102 (K102) is dependent on the intracellular AcP. The acetylation level of K102 decreases under PhoP-activating conditions including low magnesium, acid stress or following phagocytosis. Notably, *in vitro* assays show that K102 acetylation affects PhoP phosphorylation and inhibits its transcriptional activity. Both cell and mouse models show that K102 is critical to *Salmonella* virulence, and suggest acetylation is involved in regulating PhoP activity. Together, the current study highlights the importance of the metabolism in bacterial virulence, and shows AcP might be a key mediator.

## Introduction

Bacterial metabolism plays an important role in virulence [1,2]. Synthesis of many virulence factors is controlled by nutrient availability. Alternatively, central metabolites may directly regulate virulence factors activity by post-translational modification. Acetyl phosphate (AcP) is an intermediate of the phosphotransacetylase-acetate kinase (Pta-AckA) pathway [3,4]. As a high-energy form of phosphate, AcP can function as a donor of both its acetyl and phosphoryl groups, regulating protein function [4–8]. Thus, AcP can act as the phoshoryl donor to certain two-component response regulators [8] and as the acetyl donor for non-enzymatic lysine acetylation, which is considered more global in bacteria than acetyltransferase-mediated enzymatic modification [5,6]. Protein function is affected by AcP-dependent acetylation of transcription factors RcsB and CRP [9–11], isocitrate dehydrogenase and malate dehydrogenase [12,13], topoisomerase [14], Acs [15] and enolase [16]. However, the physiological role of AcP-dependent acetylation remains largely unknown.

As the regulator of the two-component system PhoP/Q, PhoP directly controls the transcription of five percent genes in *Salmonella typhimurium* [17]. PhoQ responds to low magnesium [18], low pH [19], antimicrobial peptides [20] and a decrease in the oxidizing activity of the periplasm [21] by promoting the phosphorylation of PhoP. Phosphorylated PhoP binds to promoters of target genes [22] and recruits RNA polymerase to initiate gene transcription *in vivo* [23]. PhoP can accept phosphoryl groups from both PhoQ and AcP *in vitro*, whereas it is phosphorylated by PhoQ *in vivo* [24,25].

Previously, we demonstrated that the acetylation of PhoP K201 impairs its DNA-binding ability, resulting in attenuated virulence of *S. typhimurium* [26]. Here, we show that PhoP can be regulated by AcP-dependent acetylation *in vivo* and *in vitro*. The acetylation level of PhoP K102 was positively correlated with the concentration of intracellular AcP under various conditions. Additionally, the acetylation of K102 is involved in the bacterial response to the various types of environmental stress. These findings indicate that the AcP-dependent acetylation of K102 inhibits the phosphorylation of PhoP and decreases the virulence of *S. typhimurium*, revealing the underlying mechanism of bacterial virulence regulated by primary metabolism.

## Results

### Phop can be acetylated by acetyl phosphate

**Figure 1.**
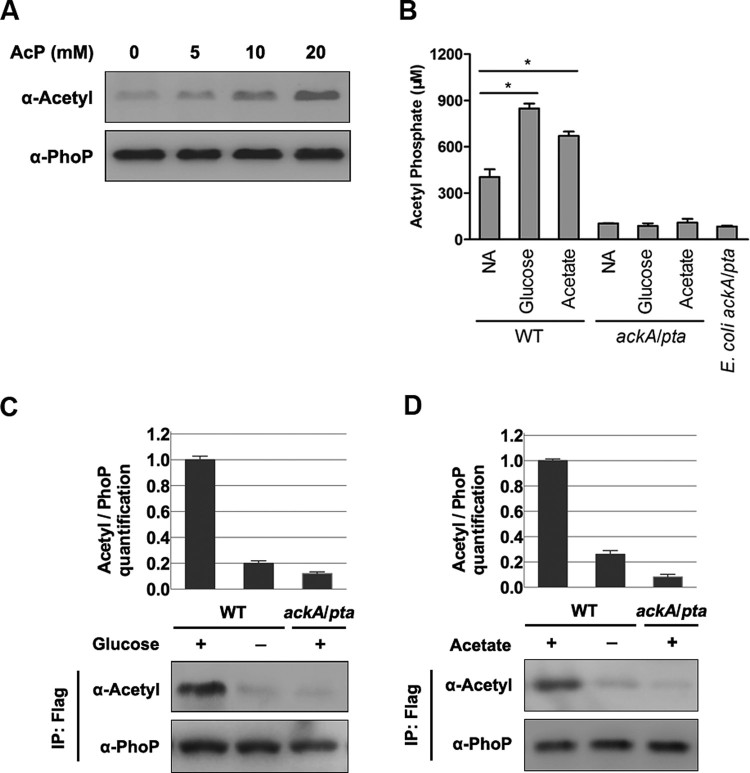
*AcP is involved in the acetylation of PhoP.* A, The acetylation level of PhoP after AcP treatment. The acetylation levels of AcP treated PhoP were detected by the pan-specific anti-acetyllysine antibody (α-Acetyl). B, The intracellular concentrations of AcP with independent glucose and acetate supplementation. Error bars show standard deviations (SDs) of triplicate measurements. C, The acetylation level of PhoP with glucose supplementation. The chromosomal *phoP-*Flag*-*tagged *S. typhimurium* (*phoP*-Flag) was cultured with or without glucose supplementation, and the *phoP*-Flag on the *ackA*/*pta* double deletion mutant background was cultured with glucose supplementation. PhoP was immunoprecipitated using the anti-Flag antibody. The acetylation level of PhoP was determined using the pan-specific anti-acetyllysine antibody (α-Acetyl). Relative acetylation level over PhoP was quantified (top panel). Error bars represent ± SD for triplicate experiments. D, The acetylation level of PhoP with acetate supplementation. The chromosomal *phoP-*Flag*-*tagged *S. typhimurium* (*phoP*-Flag) was cultured with or without acetate supplementation, and *phoP*-Flag on the *ackA*/*pta* double deletion mutant background was cultured in LB with acetate supplementation. PhoP was immunoprecipitated by anti-Flag M2 antibody. The acetylation level of PhoP was detected by the anti-acetyllysine antibody (α-Acetyl). *, *P* < .05, Student’s *t*-test.

The acetyltransferase Pat can acetylate PhoP in *S. typhimurium* [26]. However, the PhoP protein from a *pat* deletion mutant maintains strong acetylation signals, suggesting that another mechanism(s) is likely involved in the acetylation of PhoP *in vivo* in a Pat-independent manner. Because acetyl phosphate (AcP) has a crucial role in protein lysine acetylation in *E. coli* [5,6], we tested whether that the remaining acetylation signals of PhoP from the *pat* deletion mutant might be caused by intracellular AcP. The acetylation levels of PhoP increased after *in vitro* incubation with AcP in a dose-dependent manner (Figure 1(A)).

Glucose or acetate supplementation to media can increase AcP-dependent acetylation in *E. coli* [5,6,27,28]. To address whether these carbon supplements have a similar effect on PhoP acetylation, we cultured *S. typhimurium* with glucose or acetate added in LB and checked the intracellular AcP concentration and acetylation status of PhoP as well. The *ackA*/*pta* double deletion mutant derived from *E. coli* strain BW25113 was used as a negative control because this mutant is defective in the production of AcP [4,5,29–31]. In wild-type, *S. typhimurium*, AcP concentrations in cells cultured independently with glucose or acetate addition were 2.10 and 1.66 times, respectively, higher than the levels in cells cultured in regular LB. In contrast, the *ackA*/*pta* double deletion mutant had an extremely low AcP concentration, and did not respond to glucose or acetate supplementation (Figure 1(B) and S1). Next, an *S. typhimurium* strain with a chromosomally Flag-tagged PhoP (*phoP*-Flag) was constructed and cultured in LB with glucose or acetate added, and cells were harvested at the stationary phase. Acetylation levels of PhoP in strains with glucose (Figure 1(C)) or acetate (Figure 1(D)) supplementation were 5.0 times or 3.8 times, respectively, higher than those in cells isolated from regular LB. In contrast, the acetylation of PhoP in the *ackA*/*pta* double deletion mutant was barely detected even with glucose (Figure 1(C)) or acetate (Figure 1(D)) supplementation. In other words, the intracellular AcP is responsible for PhoP acetylation under the conditions tested.

### Intracellular AcP concentrations are correlated with the transcriptional activity of PhoP

**Figure 2.**
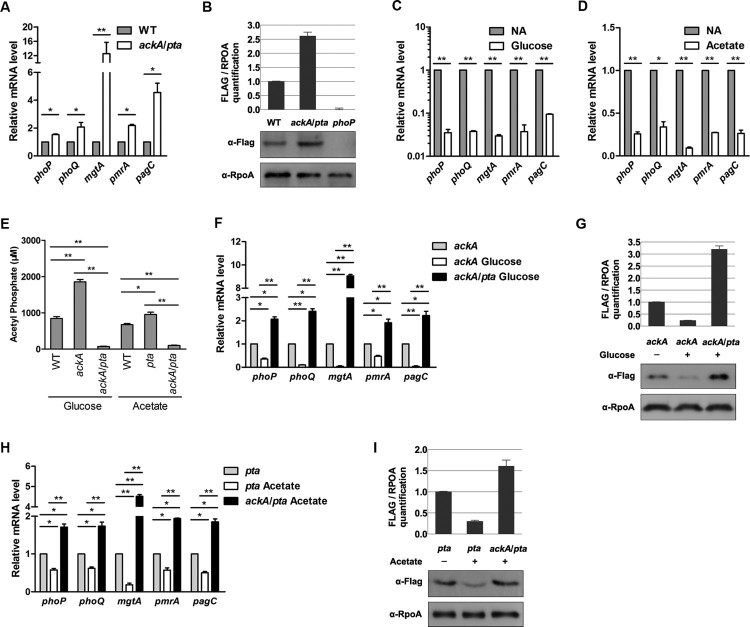
*AcP regulates the mRNA level of PhoP target genes and protein levels of PhoP.* A, Transcriptional levels of *phoP*/*Q* and downstream genes in wild-type strain and the *ackA*/*pta* mutant. B, PhoP concentration in the *ackA*/*pta* mutant strain. Relative Flag-tagged PhoP over RpoA was quantified (top panel). Error bars represent ± SD for triplicate experiments. C, The mRNA levels of *phoP*/*Q* and PhoP target genes in wild-type strain with or without glucose supplementation. Bacteria were harvested to extract RNA at the stationary phase in LB supplemented with 0.8% glucose. D, The mRNA levels of *phoP*/*Q* and PhoP target genes in wild-type strain with or without acetate supplementation. When bacteria grew to turbidity at 600 nm reached 0.4 in LB, 0.54% acetate was added and cultured for another 1 h. Then, bacteria were collected to extract total RNA. E, The intracellular concentrations of AcP in the *ackA* mutant with glucose supplementation or in the *pta* mutant with acetate supplementation. Bacteria were cultured in LB supplemented with glucose or acetate overnight and harvested for AcP measurement. Error bars indicate SDs of triplicate measurements. *, *P* < .05, Student’s *t*-test. F, The mRNA levels of *phoP*/*Q* and PhoP target genes in the *ackA* mutant. The stationary-phase bacteria in LB with or without glucose supplementation were harvested to isolate total RNA. The relative expression levels of the corresponding genes in the *ackA* mutant and *ackA*/*pta* double deletion mutant in the presence of glucose were compared with those in the *ackA* mutant without glucose supplementation (expression level set as 1). *, *P *< .05; **, *P *< .01, Student’s *t*-test. G, PhoP concentration in the *ackA* mutant. When the *phoP*-Flag strains grew to the stationary phase in LB with or without glucose supplementation, cells were harvested for western blotting. Western blot experiments were independently repeated at least three times. H, The mRNA levels of *phoP*/*Q* and PhoP target genes in the *pta* mutant. The transcriptional levels of *phoP*/*Q* and PhoP target genes in the *pta* mutant with or without acetate supplementation. When turbidity at 600 nm reached 0.4 in LB, acetate was added and cultured for another 1 h. Then, bacteria were collected to extract total RNA. *, *P *< .05; **, *P *< .01, Student’s t-test. I, PhoP concentration in the *pta* mutant. When bacteria grew to turbidity at 600 nm reached 0.4 in LB, acetate was added and cultured for another 1 h for Western blot. Western blot experiments were repeated three times. *, *P *< .05; **, *P* < .01, Student’s *t*-test.

Next, we addressed whether AcP abundance affects the physiological function of PhoP. PhoP is positively autoregulated [32]. We found that the transcriptional levels of *phoP* and other PhoP-regulated genes increased significantly in the *ackA*/*pta* double deletion mutant compared to those in the wild-type strain (Figure 2(A)). Consistent with mRNA levels, the protein levels of PhoP in the *ackA*/*pat* double deletion mutant were also 2.61 times that of the wild-type strain (Figure 2(B)). In contrast, the accumulation of AcP after glucose or acetate supplementation was associated with reduced transcription of *phoP* and PhoP-regulated genes (Figure 2(C) and (D)).

Both the *ackA* mutant cultured in LB with glucose added and the *pta* mutant cultured in LB with acetate added accumulated high levels of AcP (Figure 2(E)) as reported in *E. coli* [5,6,33]. As expected, glucose supplementation effectively down-regulated transcription of the PhoP-regulated genes (Figure 2(F)) and PhoP protein levels (Figure 2(G)) in the *ackA* mutant. Acetate supplementation had similar effects in the *pta* mutant (Figure 2(H) and (I)). In contrast, the addition with either glucose or acetate did not abolish the transcription of the PhoP/Q circuit in the *ackA*/*pta* double mutant presumably because of its defective AcP synthesis (Figure 2(F) and (H)).

### K102 is an AcP-dependent acetylation residue

**Figure 3.**
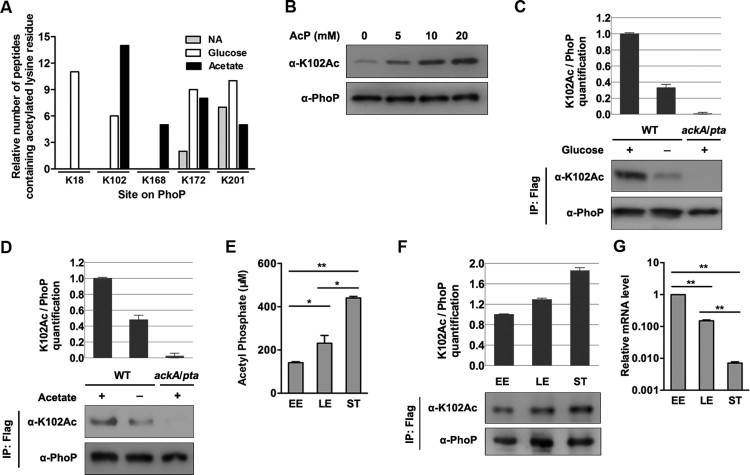
*K102 can be acetylated by AcP*. A, Relative quantification mass spectrometry measurements of acetylated lysine on PhoP with acetate or glucose supplementation. The number of peptides containing acetylated lysine residue was normalized to per 1000 trypsin-digested peptides. NA, non-addition. Experiments were performed in triplicates, and representative results were shown. B, The acetylation level of K102 after AcP treatment. The purified PhoP was incubated with different concentrations of AcP, and the acetylation level was determined using the anti-K102Ac antibody. C, The acetylation level of K102 with glucose supplementation. The chromosomal *phoP-*Flag*-*tagged *S. typhimurium* (*phoP*-Flag) was cultured in LB with or without glucose supplementation, and *phoP*-Flag in the *ackA*/*pta* double deletion mutant was cultured in LB with glucose supplementation. PhoP was immunoprecipitated using an anti-Flag antibody. PhoP acetylation level was detected with the anti-PhoP K102Ac-specific antibody. Relative K102 acetylation level over PhoP was quantified (top panel). Error bars represent ± SD for triplicate experiments. D, The acetylation level of K102 with acetate supplementation. The chromosomal *phoP-*Flag*-*tagged *S. typhimurium* (*phoP*-Flag) was cultured with or without acetate supplementation, and *phoP*-Flag in the *ackA*/*pta* double deletion mutant was cultured in LB with acetate supplementation. PhoP was immunoprecipitated using an anti-Flag antibody. K102 acetylation level was determined using the anti-K102Ac antibody. E, The intracellular concentrations of AcP at different growth phases. Bacteria cells were harvested at the indicated time points. The intracellular concentrations of AcP at early exponential phase, late exponential phase and stationary phase were measured. Error bars indicate SDs of triplicate measurements. *, *P* < .05; **, *P* < .01, Student’s t-test. F, The acetylation levels of K102 at different growth phases. EE, early exponential phase; LE, late exponential phase; ST, stationary phase. G, The transcriptional levels of *phoP* in different growth phases. The relative expression of *phoP* at LE or ST was compared to that at EE (expression level set as 1). **, *P* < .01, Student’s *t*-test analysis.

To identify the lysine residue(s) of PhoP acetylated by AcP, PhoP proteins from the wild-type *S. typhimurium* harbouring pCDSS-*phoP* cultured in LB with glucose or acetate supplementation were subjected to mass spectrometry. A total of five acetylated lysine residues (K18, K102, K168, K172, and K201) were detected (Figure 3(A)). The acetylation of K201 was detected in all three conditions in an AcP-independent manner. In contrast, the other residues (K18, K102, K168, and K172) had more acetylation detected only under AcP-rich conditions. Since K102 is the most conserved among them (Figure S2A), we focused on this residue hereafter. Interestingly, acetylation of K102 was not detected when bacteria were cultured in LB but detected in LB medium with either glucose or acetate added (Figure 3(A)). A representative mass spectrometry map of the acetylated K102 was shown in Figure S2B. To detect acetylation of K102, we prepared the anti-K102Ac site-specific antibody (Figure S3). The acetylation of K102 was detected by mass spectrometry after AcP treatment *in vitro* (Figure S2C). AcP treatment increased the acetylation level of K102 in a dose-dependent manner (Figure 3(B)). In contrast, neither Pat nor CobB was involved in regulating K102 acetylation (Figure S4A, S4B, and S4C).

The acetylation level of K102 from *S. typhimurium* cultured with glucose or acetate supplementation was 3.0 times (Figure 3(C)) and 2.1 times higher (Figure 3(D)) than that from unsupplemented LB, respectively. Consistent with the accumulated AcP level in the *ackA* mutant cultured in LB with glucose added or the *pta* mutant cultured in LB with acetate added, the K102 acetylation levels under these conditions (*ackA *+ glucose or *pta *+ acetate) were higher than in cells cultured with no supplements (Figure S4D and S4E).

### The k102 acetylation level of PhoP varies with the growth phase

We found that intracellular AcP levels increased during bacterial growth, and the concentrations of AcP at late exponential and stationary phases were 1.64 and 3.12 times, respectively, higher than that at early exponential phase (Figure 3(E)). Correspondingly, the acetylation of K102 increased gradually from early log to the stationary phase (Figure 3(F)). The transcriptional levels of *phoP* were negatively correlated with intracellular AcP concentrations (Figure 3(G)).

### The k102 acetylation level decreases under PhoP/Q-activating conditions

**Figure 4.**
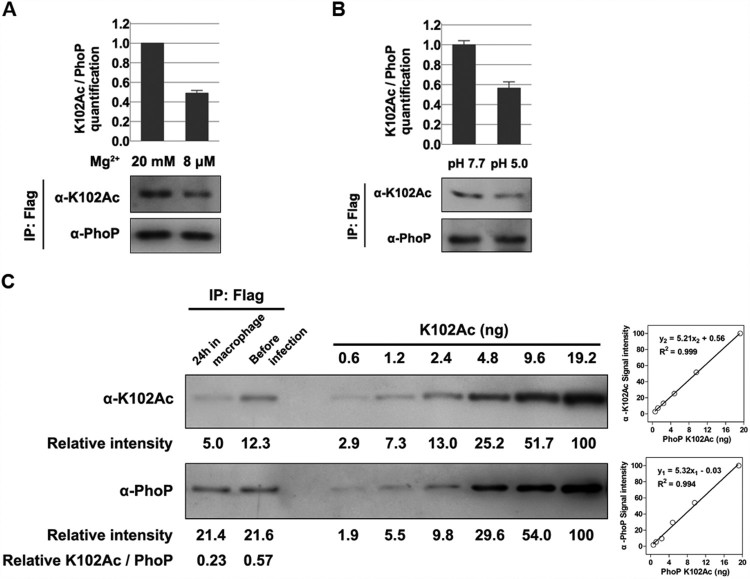
*The K102 acetylation levels under different conditions.* A, The acetylation levels of K102 with different concentrations of magnesium. Bacteria were cultured to exponential phase in LB supplemented with different amounts of magnesium. B, The K102 acetylation levels under acid stress. After transferred to EG medium at pH 5.0 or pH 7.7 for 1 h, cells were harvested for the immunoprecipitation. C, The acetylation proportion of K102 in RAW 264.7 cells. Bacteria grown overnight in LB were used to infect macrophage or harvested for immunoprecipitation as the “before infection” sample (24 h incubation). PhoP was immunoprecipitated using the anti-Flag antibody, and the acetylation proportion of K102 was determined using the anti-K102Ac antibody and the anti-PhoP in a two-fold serial dilution of K102Ac. The anti-PhoP gray value was set to “y1,” the anti-K102Ac gray value was set to “y2” and the protein content was set to “x” to build the two scatter charts. The gray values of the two samples were plotted to the standard curves, to calculate the content of total PhoP (4.11 and 4.15 ng, respectively) and K102Ac (0.96 and 2.36 ng, respectively). Lastly, the acetylation proportions of the samples were determined using K102Ac/total PhoP (23% and 57%, respectively). Western blot experiments were independently repeated three times.

We next investigated whether PhoP/Q-activating signals (such as low magnesium and intramacrophage stresses) could affect K102 acetylation *in vivo*. Bacteria were cultured to exponential phase with LB medium supplemented with different concentrations of magnesium, and the K102 acetylation level was determined via immunoprecipitated Flag-tagged PhoP. An approximately two-fold downregulation of K102 acetylation at low magnesium (8 μM) compared with a high magnesium (20 mM) concentration was observed (Figure 4(A)). Similarly, K102 acetylation levels after a mild acid treatment were analysed. Approximately 43% of the PhoP K102 acetylation signals diminished when incubated in medium at pH 5.0 (Figure 4(B)).

The two-component system PhoP/Q is crucial for *S. typhimurium* virulence, and especially for *Salmonella* survival in macrophages; hence, we evaluated the K102 acetylation level in intracellular *S. typhimurium*. Intracellular bacteria in mouse macrophage-like RAW 264.7 cells at 24-h post-infection were isolated, and PhoP was immunoprecipitated. The K102 acetylation level of the intracellular bacteria was ∼40% of that in bacteria cultured in LB (Figure 4(C)). The K102 acetylation level was positively correlated with the amount of protein in the tested protein concentration ranges using two-fold serial dilutions. The site-specific acetylation incorporated N^ϵ^-acetyllysine system was used to express PhoP K102Ac [34]. The homogeneity of K102Ac and the incorporation of acetylated K at K102 were verified (Figure S5). Thus, we calculated that the acetylated fractions of K102 under *in vitro* and *in vivo* were ∼57% and 23%, respectively (Figure 4(C)).

### K102 is essential for the phosphorylation of PhoP

K102 is located in the receiver domain and highly conserved in PhoP from both Gram-positive and Gram-negative bacteria (Figure S2A). The corresponding residue in *Mycobacterium tuberculosis* (Mtb-K121) is essential for the PhoP activity and forms a hydrogen bond to the side chain of the predicted phosphorylation residue aspartic acid (Mtb-D71) [35]. To assess the effect of K102 acetylation on PhoP phosphorylation, K102 was separately substituted to glutamine (Q), arginine (R) or alanine (A). The K-to-R substitution was not acetylated but kept a positive charge, imitating the non-acetylated form, and the K-to-Q replacement imitates a constitutively acetylated form by counteracting the positive charge [36]. Because AcP can phosphorylate PhoP *in vitro* as a donor of phosphoryl groups [37,38], AcP was used to phosphorylate wild-type PhoP and the K102 mutants. The wild-type PhoP could be phosphorylated by AcP, and phosphorylation levels increased over time (Figure S6A). In contrast, the phosphorylation band was not detected in either the K102Q, K102R or K102A variants, as well as the phosphorylation-defective variant D52A (Figure S6B).

**Figure 5.**
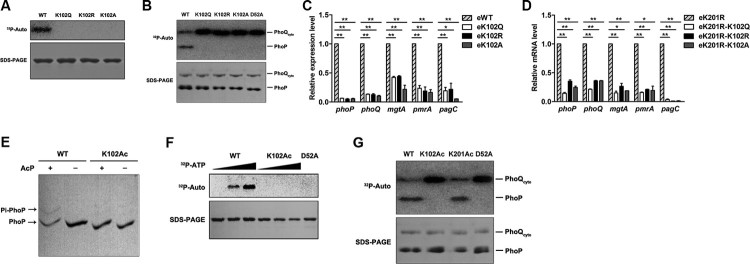
*K102 mutants and K102Ac abrogate the phosphorylation of PhoP.* A*,* Radiograph showing the phosphorylation of wild-type PhoP and K102 mutants by AcP. The PhoP WT (positive control), K102Q, K102R, and K102A were mixed with [^32^P]-AcP, and the phosphorylation was determined by overnight exposure on a phosphor-imaging screen. B, Radiograph showing the phosphorylation of wild-type PhoP and K102 mutant with phosphorylated PhoQ as the phosphoryl group donor. The upper band is the phosphorylated [^32^P]-PhoQ, and the lower band is the phosphorylated [^32^P]-PhoP. PhoQ_cyto_ was phosphorylated with [γ−^32^P]-ATP in phosphorylation buffer for 20 min at 37°C. Phosphotransfer was performed by adding the autophosphorylated PhoQ_cyto_ to wild-type PhoP or mutants in the same buffer at 37°C for 60 min. Experiments were performed in triplicates, and representative result was shown. C, The mRNA levels of *phoPQ* and downstream genes in the chromosomal *phoP* K102 mutants. D, The mRNA levels of *phoPQ* and PhoP target genes in the chromosomal *phoP* K102 mutants on the K201R background. E, The phosphorylation of K102Ac determined by the Phos-tag gel analysis. The PhoP WT and K102Ac were incubated independently with or without 50 mM of AcP in phosphorylation reaction buffer at 30°C for 2 h. The upper band is the phosphorylated PhoP, and the lower band is the unphosphorylated PhoP. F, The phosphorylation of wild-type PhoP and K102Ac by [^32^P]-AcP. D52A was used as the negative control. G, Radiograph showing the phosphorylation of K102Ac by [^32^P]-PhoQ. The indicated proteins were incubated in the phosphorylation reaction system with phosphorylated [^32^P]-PhoQ as the phosphoryl group donor. The upper band is the phosphorylated [^32^P]-PhoQ, and the lower band is the phosphorylated [^32^P]-PhoP. The SDS-PAGE serves as the loading control. *, *P* < .05; **, *P* < .01, Student’s *t*-test.

To confirm these results, we repeated the *in vitro* modification of PhoP experiment with the radioisotope AcP. Phosphorylation of native PhoP was detected, while the K102 variants could not be phosphorylated by AcP, nor the negative control D52A variant, indicating that K102 is essential for the phosphorylation of PhoP *in vitro* (Figure 5(A)). AcP can serve as a donor of both phosphoryl and acetyl groups. To minimize complications, we further used PhoQ as a phosphoryl group donor to conduct the radioisotope assay again and got consistent results (Figure 5(B)).

To avoid the artificial effect caused by overexpression, we constructed the individual chromosomal mutations of *phoP* to encode the PhoP variants K102Q, K102R, and K102A in *S. typhimurium*, which we denote as eK102Q, eK102R, and eK102A, respectively. Then, the transcriptional activity of *phoP* was determined in these eK102 mutants. As expected, the eK102Q, eK102R, and eK102A mutations significantly abolished the transcription of *phoP* and PhoP-regulated genes compared with the wild-type strain (Figure 5(C)). Acetylation of K201 prevents DNA-binding, so the unacetylatable K201R should bind DNA without hinderance. To rule out the potential impact of the acetylation of K201, transcriptional levels of these genes were measured again for the eK102Q, eK102R, and eK102A mutations in a K201R background. As shown, the transcription of *phoP* and its target genes were down-regulated in the eK201R-eK102Q, eK201R-K102R, and eK201R-K102A mutants compared with eK201R (Figure 5(D)). Moreover, mutations in *phoP* that encode K102Q, K102R, and K102A did not disturb DNA-binding of PhoP to *pho* promoter (Figure S7), which is similar to OmpR and RcsB [39,40].

### Acetylation of k102 abrogates the phosphorylation of PhoP

Site-specific acetylation facilitates the study of specific residues since it is highly efficient. The Phos-tag gel showed that native PhoP was phosphorylated by AcP, but there was no detectable phosphorylation in K102Ac (Figure 5(E)). To confirm this observation, a radioactive isotope assay was performed with [^32^P]-AcP as the phosphoryl group donor. The phosphorylation of native PhoP increased in a dose-dependent manner; however, neither K102Ac nor the negative control D52A could be phosphorylated by AcP (Figure 5(F)). To rule out a potential effect of AcP acetylation on the phosphorylation, PhoQ_cyto_ was used as the phosphoryl group donor. Using this strategy, we observed the transfer of the phosphorylation signal in native PhoP from phosphorylated [^32^P]-PhoQ_cyto_, but K102Ac could not accept the phosphoryl group. K201Ac or D52A serves as positive or negative controls, respectively (Figure 5(G)).

### K102 of PhoP is involved in *S. typhimurium* intramacrophage replication and acid tolerance responses

**Figure 6.**
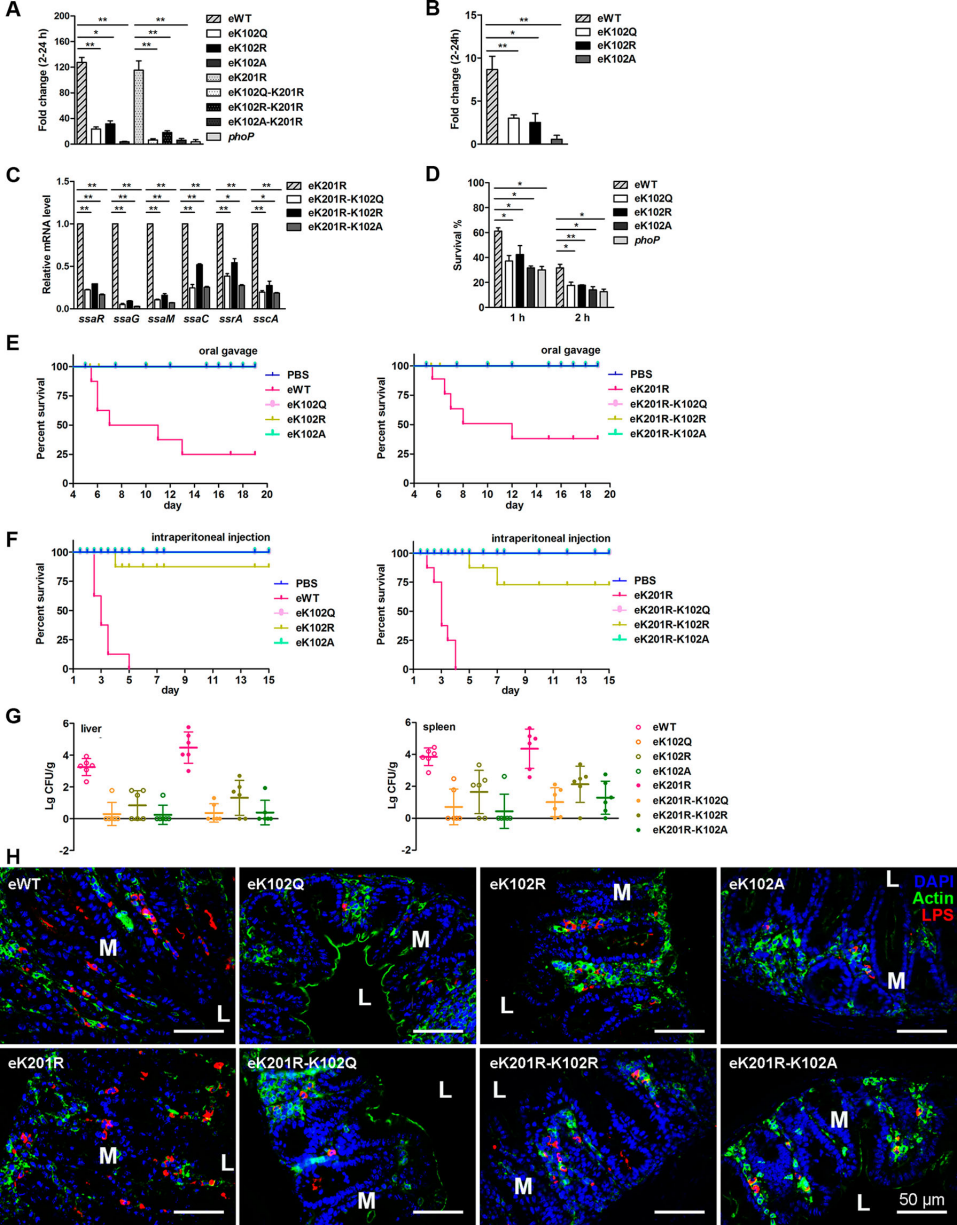
*K102 of PhoP contributes to intracellular replication and virulence in the mouse model.* A, The bacterial replication capability in mouse macrophage RAW 264.7 cells. Net growth from 2 to 24 h was analysed by the fold change in colony-forming units/ml recovered. The *phoP* deletion mutant was used as the control. B, The bacterial replication in primary peritoneal macrophages. Error bars represent SDs from three independent experiments. C, The transcriptional levels of the tested genes in the chromosomal K102 mutants on the K201R background. D, The survival rates of the chromosomal *phoP* K102 mutants in exponential phase ATR. E, Survival rates of mice infected by oral gavage. The streptomycin-pretreated mice in each group (eight mice/group) were infected by oral gavage. The number of live mice was counted twice a day. Left, the chromosomal K102 mutants; right, the chromosomal K102 mutants on the K201R background. PBS was used as the negative control. F, Survival rates of mice infected by intraperitoneal injection. Mice were assigned randomly into groups (eight mice/group) and infected intraperitoneally. Left, the chromosomal K102 mutants; right, the chromosomal K102 mutants on the K201R background. PBS was used as the negative control. G, Bacterial burdens in liver and spleen. The livers and spleens were collected 2 days after oral gavage, and colony-forming unit counting was conducted to determine bacteria numbers. Left, liver; right, spleen. H, Bacterial burdens in ceca were determined by immunofluorescence histochemical staining. Mice were euthanized by cervical dislocation 2 days after oral gavage, and the ceca were harvested. Paraformaldehyde-fixed paraffin sections were stained for actin, nuclei with 4′,6′-diamidino-2-phenylindole or Salmonella LPS. Upper is the ceca infected with the chromosomal K102 mutants, and lower is the ceca infected with the chromosomal K102 mutants on the K201R background. M, mucosa; L, intestinal lumen. *, *P* < .05; **, *P* < .01, Student’s *t*-test.

To examine the role of K102 in *Salmonella* virulence, *S. typhimurium* strains with chromosomal mutations in *phoP* that yield K102 variants were used to infect RAW 264.7 cells. The eWT proliferated by ∼125-fold from 2 to 24-h post-infection. In contrast, eK102Q and eK102R showed ∼25-fold and 32-fold increases, respectively, while eK102A only had a ∼3.5-fold replication increase, which was similar to that of the *phoP* deletion mutant (Figure 6(A)). We also determined the replication of K102 mutants in the K201R background in macrophage cells. As expected, eK201R had a 115-fold replication increase, while the replicative abilities of both e K201R-K102Q and eK201R-K102A were as low as 6-fold (Figure 6(A)). Consistent with the phenotypes in RAW 264.7 cells, the replication ability of eWT was two-fold that of eK102Q or eK102R, or 10-fold that of eK102A in mouse primary peritoneal macrophage cells (Figure 6(B)).

Because SPI-2 is essential for *S. typhimurium* intramacrophage replication, the mRNA levels of SPI-2 genes in the mutants that produce K102 variants in a K201R background were determined. The transcriptional levels of representative SPI-2 genes including *ssaR*, *ssaG*, *ssaM*, *ssaC*, *ssrA,* and *sscA*, in eK201R-K102 mutants were much lower compared with that of the eK201R single mutant (Figure 6(C)). Thus, K102 is crucial for PhoP-mediated intramacrophage replication, irrespective of macrophage types.

The acid tolerance response (ATR) assay showed that the survival rates of eK102Q, eK102R, and eK102A at 1 or 2 h were similar to these of the *phoP* deletion mutant and were significantly lower than that of eWT (Figure 6(D)). Consistently, the mutants that produce K102 variants in the K201R background possessed lower survival rates compared with eK201R (Figure S8A).

### K102 of PhoP contributes to *S. typhimurium* virulence in the mouse model

To explore the role of K102 in *S. typhimurium* virulence, we infected mice with a series of eK102 derivatives either through the oral route or intraperitoneal injection. For oral infections, eWT-infected mice began to die at 5 d post-challenge. By 13 d after infection, only 25% of the eWT-infected mice remained alive, but all of the eK102 mutant-infected mice were healthy and lively (Figure 6(E)). Next, mice were infected by intraperitoneal injection and monitored over a 15-d period. As expected, eWT displayed a strong virulence, and the mice in this group began dying 2.5 d post-challenge. All of the mice were dead by 5 d after infection. In contrast, there were no deaths in the eK102Q- or eK102A-infected mice, like the PBS group of mice, throughout the whole experimental period, and one eK102R-infected mouse died on day 4 post-infection (Figure 6(F)). The K102 mutants on the K201R background showed similar results in the mouse model, except that two eK201R-K102R-infected mice died on day 5 and day 7 after an infection following intraperitoneal injection (Figure 6(E) and (F)). The bacterial burden of eWT-infected mice was much greater than those of eK102R, eK102Q, and eK102A in the spleen and liver. A similar experiment was performed using the K102 mutant on the K201R background. There were approximately 10^4^ colony-forming units of eK201R per gram of tissue, which was about 100-fold that of eK201R-K102R and 1000-fold that of eK201R-K102Q and eK201R-K102A in both spleen and liver (Figure 6(G)).

Furthermore, both eWT and eK201R colonized the caecum, and eK102R and eK201R-K102R were partially detected in ceca. However, eK102Q, eK102A, eK201R-K102Q, and eK201R-K102A were barely detected in ceca (Figure 6(H)). Furthermore, desquamation in the superficial epithelium layer was apparent in eWT- and eK201R-infected mice, especially at the infection site, followed by eK102R and eK201R-K102R-infected mice, while nearly no desquamation was observed in eK102Q-, eK102A-, eK201R-K102Q-, and eK201R-K102A-infected mice (Figure S8B). Other phenomena, including polymorphonuclear leukocyte infiltration, oedema, and the loss of goblet cells, frequently occurred in eWT- and eK201R-infected ceca, indicating strong intestinal inflammation. In contrast, intestinal inflammation was hardly detected in eK102Q-, eK102A-, eK201R-K102Q-, and eK201R-K102A- infected mice ceca, and was alleviated in eK102R- and eK201R-K102R-infected mice (Figure S8C).

## Discussion

### Crosstalk between acetylation and phosphorylation

In this study, we demonstrate that the acetylation of K102 of PhoP inhibits the phosphorylation of PhoP, which significantly reduces *Salmonella* virulence by downregulating the transcriptional activity of *phoP*. In eukaryotes, there are often multiple sites of covalent modification on a single protein, and crosstalk among the PTMs occurs universally [41,42]. In bacteria, to our knowledge, the only example of crosstalk between two PTMs has been observed in CheY, a response regulator of the chemotaxis system in *E. coli* [43,44]. CheY is the first response regulator identified to be acetylated [45]. Several acetylated lysine residues have been identified on CheY, leading to diverse effects, including rotational direction of flagella, and formation of flagellar switch complex [43,44,46–50]. One important acetylation site is K109 [46,51], which is linked in co-regulation between CheY phosphorylation and acetylation [43], and this lysine residue (corresponding residue K102 on PhoP) is highly conserved in response regulators [52].

Here, we show that the acetylation levels of PhoP K102 under different culture conditions and stresses correlate with PhoP activities. *M. tuberculosis* PhoP K121 (corresponding residue of *S. typhimurium* K102) is essential for the PhoP activity and forms a hydrogen bond with the side chain of the predicted phosphorylation residue aspartic acid D71 (corresponding residue of *S. typhimurium* D52) [35]. Combining this and the above data, we conclude that the acetylation on K102 plays an important role in regulating PhoP activity by abrogating its phosphorylation. Neither K102Q nor K102R, which mimic deacetylation and acetylation, could be phosphorylated *in vitro*. The Q and R substitutions may only mimic the charge of acetylated- or non-acetylated lysine, while the lengths of their side chains are different. All of the variants, including K102Q, K102R, K102A, and K102Ac, were predicted to abrogate the salt bridge and cause conformational rearrangements in the loops between helices and β-strands.

### AcP-dependent acetylation of PhoP k102

As a phosphoryl donor, AcP is not only responsible for a substantial amount of ATP during fermentation, but for the regulation of several response regulators in various bacterial species. For example, AcP activates capsule formation and inhibits flagellar biogenesis mediated by phosphorylation [8,53]. As an acetyl donor, non-enzymatic acetylation by AcP is thought to be less specific and more global compared with enzymatic acetylation [6]. Acetylation studies in *E. coli* revealed a critical role of AcP in protein lysine acetylation [5]. AcP has been implicated in controlling RcsB function [9] and regulates enolase activity *via* acetylation [16]. However, the role of AcP in bacterial physiology is not completely understood. Although AcP could phosphorylate PhoP *in vitro*, phosphorylation of wild-type PhoP did not rely on AcP *in vivo* [24,25]. Here, transcriptional levels of *phoP* and its target genes enhanced in AcP synthesis defective strain. In contrast, under AcP accumulating conditions (the wild-type strain with glucose or acetate supplementation, the *ackA* mutant with glucose addition, the *pta* mutant with acetate addition, and stationary-phase culture), the transcription of *phoP* and its downstream genes were repressed. Thus, there is an inverse/negative correlation between intracellular AcP level and PhoP activity. Based on mass spectrometry, K102 acetylation was identified as being AcP-dependent because it was over-represented in bacteria during growing in LB supplemented with glucose or acetate, as well as after AcP treatment of PhoP *in vitro*. Additionally, we failed to detect the K102 acetylation signals of PhoP from the *ackA*/*pta* double deletion mutant even in the presence of glucose or acetate supplements. These findings suggest that AcP regulates *phoP* regulon through the acetylation of PhoP in *Salmonella*.

AcP is an unstable phosphorylated molecule in the cell [30,31], and its concentration depends on the nature and quantity of the carbon source relative to the availability of phosphate, oxygen, and the status of the TCA cycle [4]. Other environmental factors can also impact the concentration of AcP, including pH, the concentration of extracellular acetate and temperature [54]. AcP levels have been measured in *E. coli* [5,30,31,33] but are still unknown in *Salmonella*. Here, we determined that glucose and acetate supplementation significantly elevated AcP levels, which is consistent with observations in *E. coli* [4]. AcP accumulates in growth-arrested *E. coli* cells in M9 medium with or without glucose added [5]. Acetyl phosphate-dependent acetylation is a response to carbon flux that could regulate central metabolism [27]. Supplement magnesium in TB7 buffered tryptone broth with glucose (TB7-glucose) media promotes consumption of glucose by extending the exponential phase of growth, resulting in decreased AcP concentration and lower protein acetylation [55]. We also observed that intracellular AcP progressively increased with the bacterial growth phase. During growth on media supplemented with a sugar, cultures of bacteria entered stationary phase prior to complete consumption of the sugar. The majority of the sugar was consumed after the culture stopped growing. Protein acetylation occurs primarily during the stationary phase when acetyl phosphate, the major acetyl donor in *E. coli* (and almost certainly in *Salmonella*), is produced as an intermediate of the acetate fermentation pathway [55]. However, the AcP level peaks at an early stage of exponential growth in TB medium in *E. coli* [31]. It is due to a difference in the growth conditions. Pruss and Wolfe used TB with no added sugar [55], whereas Kuhn *et al.* added glucose to the TB [6]. In *E. coli*, glucose does not catabolite repress the consumption of several easily metabolized amino acids [55]. In TB plus glucose or LB plus glucose, the culture enters stationary phase before consuming the majority of the glucose; thus, AcP accumulates in stationary phase and not in exponential phase (as it does when glucose is not added to TB or LB).

### The coordinated regulation of different acetylation residues on PhoP

**Figure 7.**
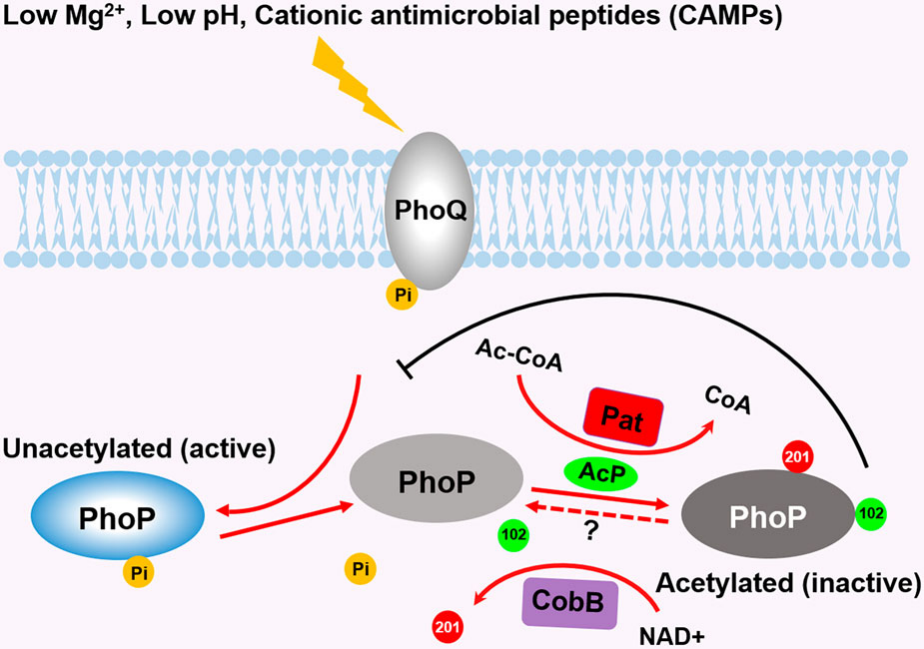
*Crosstalk between acetylation and phosphorylation on PhoP.* Under low magnesium or low pH conditions, PhoP is activated by accepting phosphoryl groups from PhoQ. The phosphorylated PhoP antagonizes the acetylation of K102. K102 is acetylated by AcP, leading to the abrogation of PhoP phosphorylation, while the acetylation of K201 by the acetyltransferase Pat inhibits the DNA-binding ability of PhoP. The deacetylation of K201 by CobB enhances the DNA-binding activity of PhoP. The deacetylation of K102 by unknown mechanisms may produce the naïve PhoP, the counterpart of PhoQ. Abbreviations: 102, K102 acetylation; 201, K201 acetylation; Pi, phosphorylation; Mg^2+^, magnesium; Ac-CoA, Acetyl-CoA; CoA, coenzyme A.

The acetylation of K201 located in the C-terminus inhibits the DNA-binding ability of PhoP [26]. Here, we illustrated acetylation at K102 abolished the phosphorylation of PhoP. Thus, acetylation can regulate PhoP activity in multiple ways. The acetyl group of K102 is transferred from AcP, while the acetyl group of K201 is derived from Ac-CoA. Considering the strong correlation between Ac-CoA and AcP concentrations during growth [30,31], it is likely that the acetylation of K102 and K201 synergistically coordinates the activity of PhoP. Under PhoP/Q-activating conditions, acetylation of both K102 and K201 is down-regulated. The unacetylated K102 provides a positively charged microenvironment to stabilize the phosphorylation of PhoP, and the deacetylated K201 could further promote DNA-binding. Additionally, the independent regulation of phosphorylation and DNA-binding also guarantees the activation of transcription in a phosphorylation-independent manner [38]. Eventually, a decrease in acetylation of these lysine residues results in a surge in PhoP activity [32] and facilitates *Salmonella* growth under stress, intramacrophage survival, and virulence (See model, Figure 7). *Salmonella* may sense various environmental stresses and regulate intracellular AcP synthesis to control its protein acetylation. Meanwhile, AcP accumulation is a consequence of fermentation, and the cells regulate whether they enter acetate fermentation. Therefore, the AcP accumulation and subsequent AcP-dependent acetylation is a result of that fermentation. As fermentation is a consequence of carbon overflow (caused by low oxygen, low phosphate, low nitrogen, or low oxygen concentrations relative to carbon), AcP-dependent acetylation could be a response to carbon overflow, with acetylation of some enzymes helping to relieve the stresses of carbon overflow [28]. In fact, all the AcP concentrations measured by ATP conversion method are the average of intracellular AcP concentrations. Perhaps, AcP may accumulate in a certain region of cell (*e.g.* membrane region) and reach a very high concentration. The second possibility for cells to control AcP-dependent protein acetylation is by deacetylation enzymes. However, only very few lysine deacetylases (KDACs) have been identified in prokaryotes, and CobB is the only one in *Salmonella*. The third reason is reduced fermentation may cause less AcP available to donate acetyl groups to proteins. For example, magnesium supplementation prolongs the exponential phase of growth. During this phase, acetylated proteins are constantly diluted by the synthesis of nascent unmodified proteins and cell growth. In stationary phase, when acetylation would normally accumulate, little carbon remains to be fermented and less acetyl phosphate is available to donate acetyl groups to proteins [55].

### K102 acetylation contributes to *Salmonella* virulence

Glutamine or arginine substitution is usually used to mimic acetylated or non-acetylated lysine residues, respectively [56, 57]. In the mouse model using intraperitoneal injection, one eK102R-infected mouse and two eK201R-K102R-infected mice died, while eK102Q-infected mice survived the infection. Moreover, the bacterial burdens in livers and spleens of eK102R-infected mice were greater than those of eK102Q-infected mice regardless of the strain’s genetic background. Immunohistochemical assays also showed similar trends: K102R mutants on either eWT or eK201R background could colonize ceca to a higher level compared with K102Q mutants. Therefore, mutation of K102R may still maintain a partial phosphorylation of PhoP *in vivo.* Instead, K102Q had more severely compromised phosphorylation and led to nearly complete loss of virulence. The failure to detect the phosphorylation of K102 derivatives *in vitro* may be due to the cellular microenvironment is different enough to permit the phosphorylation as well as the low sensitivity of assays.

Acetylation is a mechanism to modify the side chains of lysine residues and regulate protein activities, and protein acetylation is known to play an important role in bacterial virulence [58]. Therefore, *Salmonella* may change its acetylation status in response to different stimuli, which ultimately changes the phosphorylation of PhoP and its transcriptional activity. Presently, it is impossible to establish a direct link between the acetylation of one specific lysine residue and protein function using cell or animal models due to technical limitations. However, the evidence is accumulating that acetylation acts as a fine-tuning switch to control the activities of PhoP and subsequently mediate *Salmonella* virulence.

## Materials and methods

### Plasmids, strains, primers, and antibodies

All plasmids, strains, primers, and antibodies used in this study were listed in Supplemental Table 1, Table 2, and Table 3, respectively.

### Media

Lysogeny broth (LB) was used as a rich medium, supplemented with 0.8% (W/V) glucose or 0.54% (W/V) acetate as indicated. The minimal E glucose (EG) medium contained MgSO_4_ (0.098 g/L), citric acid monohydrate (2.0 g/L), K_2_HPO_4_ ·3H_2_O (13.1 g/L), NaNH_4_HPO_4_ (2.29 g/L), pH 7.7, supplemented with 0.4% (W/V) glucose (pH was adjusted with HCl). Agar plates contained 1.5% (W/V) agar, and medium was supplemented with antibiotics as required. The following antibiotics were used at the concentrations indicated; ampicillin, 100 μg/ml; chloramphenicol, 17 μg/ml; kanamycin, 50 μg/ml and spectinomycin, 50 μg/ml.

### Purification of PhoP and derivatives

Plasmids producing PhoP, K102R, K102Q, K102A, or D52A were transformed into BL21 (DE3) individually. IPTG (0.5 mM) was added when the turbidity at 600 nm reached 0.6, followed by incubation at 25°C for 4–5 h. Cells were harvested by centrifugation, and stored at −80°C for further purification. For *in vitro* acetylation, pCDSSara-*phoP* was transformed into the *Salmonella ackA/pta* mutant or *pat* mutant to express PhoP with low acetylation levels. Arabinose (10 mM) was added when the seed culture was diluted 1:100 and bacteria cells were harvested when turbidity at 600 nm reached 0.6. The purification of PhoP and its variants was performed as described previously [26]. The polypeptide compositions of the column fractions were monitored by 12% SDS-PAGE, and subsequent staining with Coomassie bright blue.

### AcP-mediated acetylation assay *in vitro*

The AcP-mediated acetylation reaction *in vitro* was performed as described previously [5]. The buffer contained 20 mg/ml BSA, 150 mM NaCl, 50 mM Tris-HCl (pH 7.5), and 1 mM EDTA. PhoP or derivative (0.1 μg/μl) was mixed with a different concentration of AcP as indicated at 37°C for 2 h.

### Quantitative reverse transcription real-time PCR (qRT-PCR) assay

Bacterial cells were cultured in EG medium at pH 7.7 and harvested when turbidity at 600 nm reached 0.4. Bacteria were lysed by a Beadsbeater, and total RNA was isolated using Trizol (Life Technology) as previously described [26].

### Acid tolerance response (ATR)

ATR was performed as previously described [26]. Briefly, bacteria were cultured in EG medium at pH 7.7 until turbidity at 600 nm reached 0.4, and then transferred to pH 5.8 EG medium for 1 h before transferred to pH 3.3 acid challenge. Cfu counting was applied for bacteria at 0, 1, and 2 h post-acid challenge. The data shown represent the average percentage of survival. Each experiment was performed three times.

### The anti-PhoP K102Ac-specific polyclonal antibody preparation

The synthesized immune peptide LSSGADDYVTK(Ac)PFH was used as an antigen to immunize rabbits. Rabbits were immunized for four times in two months, and the antisera were collected for further purification. The control peptide LSSGADDYVTKPFH was used to remove non-specific antibody. Antibody specificity and sensitivity were evaluated by ELISA and Western blot.

### Expression site-specifically acetylated PhoP (K102Ac) in *E. coli* strain BL21

Two plasmids, pAcKRS-3 and pCDF-PylT-*phoP* K102 (TAG), were co-transformed into *E. coli* strain BL21 to produce site-specifically acetylated PhoP K102Ac. The transformant was grown overnight with kanamycin and spectinomycin (LB-KS) (25 and 100 mg/ml, respectively). When the culture reached the mid- exponential phase (turbidity at 600 nm ∼1.8), the culture was mixed 1:1 with LB buffered 20 mM acetyllysine (final pH 7.2). Simultaneously, 0.5 mM IPTG was added, followed by cultured at 30°C for an additional 6 h, then harvest cells for further purification.

### Expression and purification of PhoQ_cyto_

For the purification of the His-tagged PhoQ cytoplasmic domain (PhoQ_cyto_), *E. coli* strain BL21 cells transformed with the pQE80-*phoQ*_cyto_ plasmid were grown at 37°C in 500 mL LB supplemented with ampicillin. IPTG was added at a final concentration of 1 mM when turbidity at 600 nm arrived at 0.6, followed by incubation at 37°C for 4 h. Cells were harvested and lysed as described above and the supernatant was loaded on a 1 ml Ni-NTA column (GE Healthcare, USA). The column was washed initially with Binding buffer (20 mM Tris-HCl pH 7.6, 500 mM NaCl, 20 mM imidazol), and eluted with 2 ml aliquots of Binding buffer containing 20, 40, 60, 80, or 100 mM imidazole, respectively. Protein purity was assessed to be >90% on the basis of SDS-PAGE.

### Phosphorylation assay of PhoP by the radioactive isotope

[^32^P]-AcP was produced using *E. coli* acetate kinase (Sigma) and [γ−^32^P]-ATP [59]. To obtain [^32^P]-AcP, 0.2 U of acetate kinase (Sigma Aldrich) and 200 μCi of γ-ATP (6000 Ci/mmol, Perkin Elmer) were buffered in 25 mM Tris-HCl (pH 7.4), 60 mM NaAc, 10 mM MgCl_2_ at 25°C for 30 min. 4 μg of PhoP or its derived mutants were incubated with [^32^P]-AcP at 30°C for half an hour. The reactions were stopped by adding Sample buffer. Samples were separated by SDS-PAGE gels, and gels were exposed 12 h on a phosphor screen (GE Healthcare). The signal was visualized with the Fujifilm FLA7000 imager.

Phosphorylation of PhoP by PhoQ_cyto_ were performed as follows: 1 μg of the PhoQ_cyto_ protein was phosphorylated with 10 μCi of [γ−^32^P]-ATP in 50 μl of phosphorylation buffer (50 mM Tris-HCl (pH 7.5), 100 mM KCl, 0.5 mg/ml BSA, 1 mM MgCl_2_), for 20 min at 37°C. Phosphotransfer was performed by adding the autophosphorylated PhoQ_cyto_ to 2 μg of the WT PhoP or mutants in the same buffer. The reaction was carried out at 37°C for 1 h and stopped by adding Sample buffer.

### Phosphorylation of PhoP by AcP

The phosphorylation of PhoP by AcP was performed as described [60]. Briefly, PhoP was added to phosphorylation buffer containing 50 mM HEPES (pH 7.5), 100 mM NaCl, 50 mM AcP, and 10 mM MgCl_2_, and the mixtures were incubated at 37°C for 1 h or as indicated. The reaction was stopped by addition of Sample buffer and further incubation at room temperature for 10 min (the phosphoryl group on aspartic acid is heat labile). The phosphorylation of PhoP was detected by the Phos-tag gel.

### Cell infection model

Mouse macrophage-like RAW 264.7 cells were grown in DMEM with 10% fetal bovine serum at 37°C and 5% CO_2_. Infection experiments were performed as described [26]. Briefly, cells were seeded in 24-well plates (2.5 × 10^5^ per well). Bacteria cells were diluted to achieve a multiplicity of infection (MOI) of 10. The plates were centrifuged at 400 g for 5 min at 25°C to increase phagocytosis and then incubated for 30 min in 5% CO_2_. Extracellular bacteria were removed by washing twice with DMEM, and then infected RAW 264.7 cells were incubated in fresh medium with 100 μg/ml gentamicin for 2 h. The medium was substituted by fresh medium plus 15 μg/ml gentamicin for the rest of the process. To measure the bacterial quantity in the macrophages, infected cells were lysed with Triton X-100. The number of viable intracellular bacteria was calculated via series dilutions and plating. All experiments were performed in triplicate.

Thioglycolate-elicited primary peritoneal macrophages (Peritoneal MΦ) were collected as previously described [26]. BALB/c mice were intraperitoneally vaccinated with 4% Brewer thioglycolate medium (Sigma). Mice were sacrificed after three-day treatment, and cells were acquired by flushing the peritoneal cavity with PBS. The cells were seeded in 24-well dishes and cultured overnight. The subsequent infection protocol was similar to RAW 264.7 cells.

### Animal studies

Eight-week-old BALB/c female mice were used in this study. Mice were divided into indicated groups randomly (eight mice each group). For oral infection, mice were deprived of food and water for 4 h prior to feeding streptomycin (20 mg) by oral gavage. 20 h after streptomycin treatment, food and water were withdrawn again for 4 h. Then, 1.5 × 10^7^ cfu bacteria in 100 μl PBS were fed, and the control mice were given 100 μl PBS. For intraperitoneal injection, 1.5 × 10^5^ cfu bacteria were injected, and control mice were given 100 μl PBS. Dead mice were counted twice a day.

### Ethics statement

The mice tests were approved by Shanghai Jiao Tong University School of Medicine, and these tests were performed with strict observance of the National Research Council Guide for Care and Use of Laboratory Animals [SYXK (Shanghai 2007-0025)].
